# Cortical Synchrony as a Mechanism of Collinear Facilitation and Suppression in Early Visual Cortex

**DOI:** 10.3389/fnsys.2021.670702

**Published:** 2021-07-29

**Authors:** Kris Evers, Judith Peters, Mario Senden

**Affiliations:** ^1^Department of Cognitive Neuroscience, Faculty of Psychology and Neuroscience, Maastricht University, Maastricht, Netherlands; ^2^Maastricht Brain Imaging Center (M-BIC), Maastricht University, Maastricht, Netherlands; ^3^Department of Vision and Cognition, Netherlands Institute for Neuroscience, Royal Netherlands Academy of Arts and Sciences (KNAW), Amsterdam, Netherlands

**Keywords:** collinear facilitation and suppression, cortical oscillations, neural synchrony, Kuramoto model, visual cortex, surround modulation

## Abstract

Stimulus-induced oscillations and synchrony among neuronal populations in visual cortex are well-established phenomena. Their functional role in cognition are, however, not well-understood. Recent studies have suggested that neural synchrony may underlie perceptual grouping as stimulus-frequency relationships and stimulus-dependent lateral connectivity profiles can determine the success or failure of synchronization among neuronal groups encoding different stimulus elements. We suggest that the same mechanism accounts for collinear facilitation and suppression effects where the detectability of a target Gabor stimulus is improved or diminished by the presence of collinear flanking Gabor stimuli. We propose a model of oscillators which represent three neuronal populations in visual cortex with distinct receptive fields reflecting the target and two flankers, respectively, and whose connectivity is determined by the collinearity of the presented Gabor stimuli. Our model simulations confirm that neuronal synchrony can indeed explain known collinear facilitation and suppression effects for attended and unattended stimuli.

## 1. Introduction

Stimulus-induced cortical oscillations in the gamma range are ubiquitous in visual cortex (Bertrand and Tallon-Baudry, 2000; Buzsáki and Wang, 2012; Brunet et al., 2015). The functional role of gamma had, however, been called into question on the grounds that the precise frequency of the gamma rhythm strongly depends on stimulus features (Ray and Maunsell, 2010; Jia et al., 2013). Recently, a series of studies looking at local gamma from the perspective of *weakly coupled oscillators* (WCOs) have shown that it may be precisely this feature-dependence of frequency in conjunction with both distance and feature dependence of lateral connectivity among topographically organized neuronal groups that endows gamma with its functional power (Lowet et al., 2015, 2017, 2018). According to this account, perceptual integration and segregation of stimulus elements is the functional correlate of the success and failure, respectively, to synchronize across neuronal groups coding these elements.

Generally, synchronization among WCOs depends on their frequency differences (detuning). Oscillators exhibiting more similar frequencies synchronize more readily with one another (Kuramoto, 1984; Acebrón et al., 2005). In addition, synchronization among WCOs depends on the strength of their coupling. As coupling strength increases, synchronization can be achieved across wider frequency gaps (Acebrón et al., 2005). In light of this, several empirical observations suggest that WCOs may underlie cortical processes: (1) local stimulus features determine gamma frequency in neuronal groups (Henrie and Shapley, 2005; Roberts et al., 2013; Shapira et al., 2017) (see also the empirically constrained oscillator model by Zachariou et al., 2021), (2) lateral connectivity is distance-dependent (Gilbert and Wiesel, 1989; Harris and Mrsic-Flogel, 2013) and (3) neural populations representing similar features are grouped in cortex (e.g., retinotopy, Bonhoeffer and Grinvald, 1991; Blasdel, 1992; Tootell et al., 1998; Wandell et al., 2005). These findings provide the neurophysiological and anatomical prerequisites for synchronized neural populations to represent similar, proximal image elements. Similarly, neural populations that represent dissimilar, distal, image elements fail to synchronize. A number of studies have shown that these principles can provide a mechanistic account of perceptual phenomena such as contour integration (Li, 1998), texture discrimination (Baldi and Meir, 2008) and figure-ground segregation (Yamaguchi and Shimizu, 1994). We argue that the same mechanism may also underlie collinear facilitation and suppression effects.

Collinear facilitation (or suppression) is the phenomenon that the detectability of a target Gabor is improved (or diminished) by the presence of collinear flanking Gabors (Polat and Sagi, 1993; Kapadia et al., 1995; Zenger and Sagi, 1996). At the neurophysiological level, this has been linked to the observation that firing rates of V1 neurons in response to the target presented in their “classical” receptive fields (RF) can be enhanced (suppressed) by collinear flankers placed within their non-classical receptive fields (Kapadia et al., 1995; Polat et al., 1998; Mizobe et al., 2001). Some key factors affecting facilitation and suppression are the contrast of the flankers, the global alignment of the target and flanker orientation and whether flankers are attended or ignored (Polat and Sagi, 1993; Polat et al., 1998; Freeman et al., 2001, 2003). Polat et al. (1998) showed, for instance, that the same flanker-target configuration can induce both facilitation and suppression, depending on stimulus contrast. In their study, responses of V1 neurons representing the target were facilitated if the target contrast was low compared to the contrast of the flankers and suppressed if the target contrast was high.

**Figure 1:**
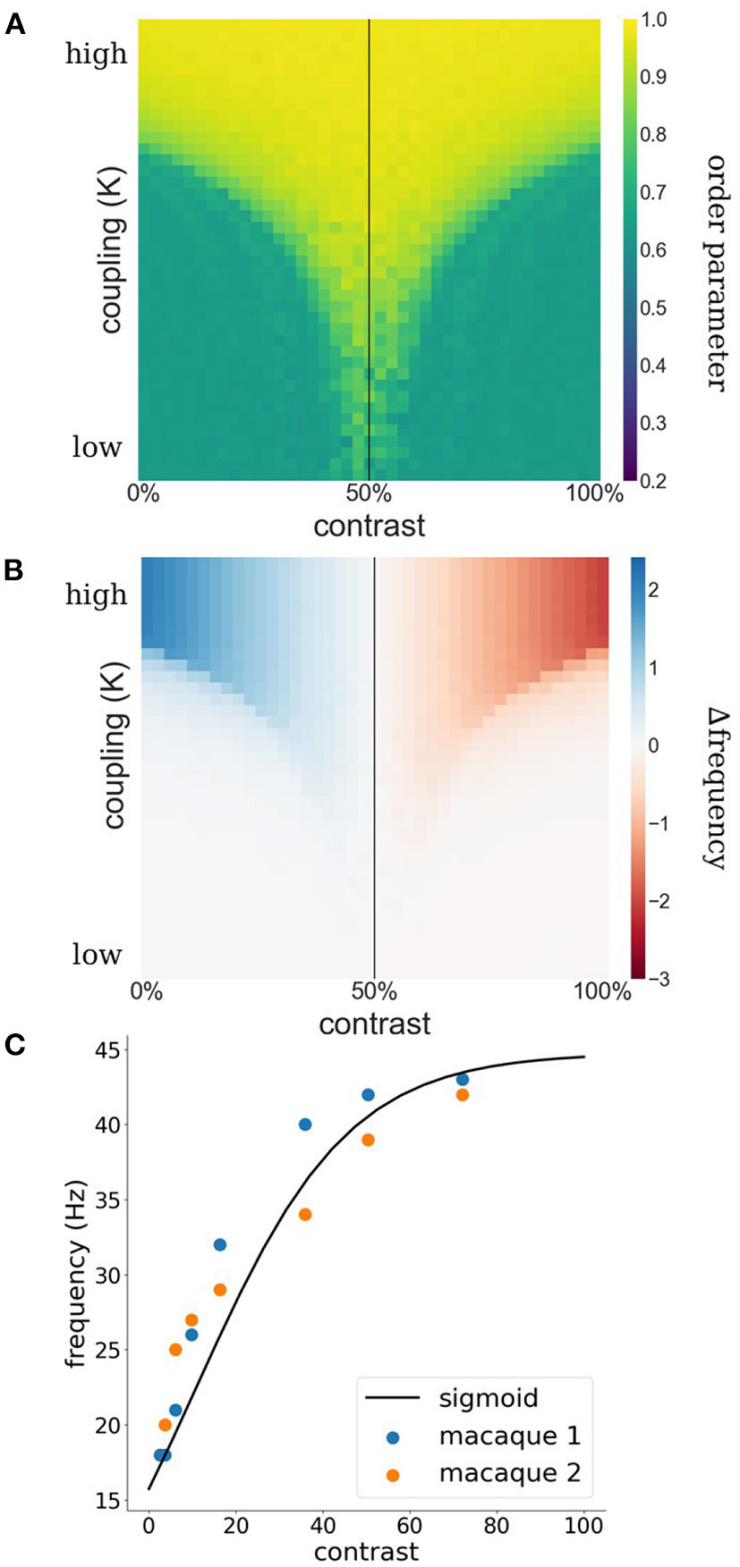
**(A)** Phase coherence between two WCOs. The contrast input to the first, reference, oscillator is fixed at 50% (black vertical line) whereas the contrast input to the second oscillator varies (x-axis) leading to detuning between their intrinsic frequencies. Coupling strength between the two oscillators varies along the y-axis. A tongue-shaped region (yellow) indicates where the pair of oscillators synchronizes. **(B)** Difference between observed (mean effective frequency) and intrinsic frequency of the reference oscillator. Within the synchronization region, the reference exhibits an increased frequency when its intrinsic frequency is lower than that of the second oscillator (blue) and a decreased frequency when its intrinsic frequency is lower than that of the second oscillator (red). **(C)** Estimation of contrast-frequency relationship from electrophysiological data. Colored dots represent peak frequency of empirically measured LFPs as a function of stimulus contrast (blue and orange dots represent data of two individual monkeys). The solid line reflects the best fitting sigmoid curve.

Such a switch from facilitation to suppression may appear surprising at first. However, it is a natural consequence of a synchronization mechanism. Synchronization is the result of lateral interactions among WCOs which causes adjustments in their effective frequency away from their intrinsic frequencies determined by feedforward processing. Specifically, oscillators with low frequencies need to *speed up* while oscillators with high frequencies need to *slow down*, a process generally referred to as entrainment. In Figures 1A,B the concept of entrainment is visualized based on two interacting Kuramoto WCOs. The Kuramoto order parameter (see Equation 3) is used as a measure for phase-coherence among the WCOs. As can be appreciated from the figure, there exists a lingulate region, the so-called Arnold tongue, in the parameter space spanned by contrast and coupling strength wherein the WCOs synchronize. Figure 1B shows the entrainment of a reference oscillator which is coupled to another oscillator with fixed input. The strength of the entrainment is dependent on relative input to the oscillators and the coupling of the oscillators.

When a target Gabor is flanked by collinear Gabors with a lower (or higher) contrast, neuronal populations encoding the target exhibit a higher (or respectively lower) intrinsic frequency than the populations representing the flankers. Through mutual interactions between target and flankers that lead to their synchronization, the effective frequency of the target population will increase (decrease), thereby leading to the observed facilitation and suppression effects. The contrast dependence of intrinsic frequencies further raises the possibility that attention effects on collinear facilitation result from attention-related increases of the contrast response in early visual cortex (Gandhi et al., 1999; Somers et al., 1999). We thus propose that collinear facilitation and suppression effects result from interactions among WCOs whose intrinsic frequencies are jointly determined by stimulus contrast and attention.

**Figure 2:**
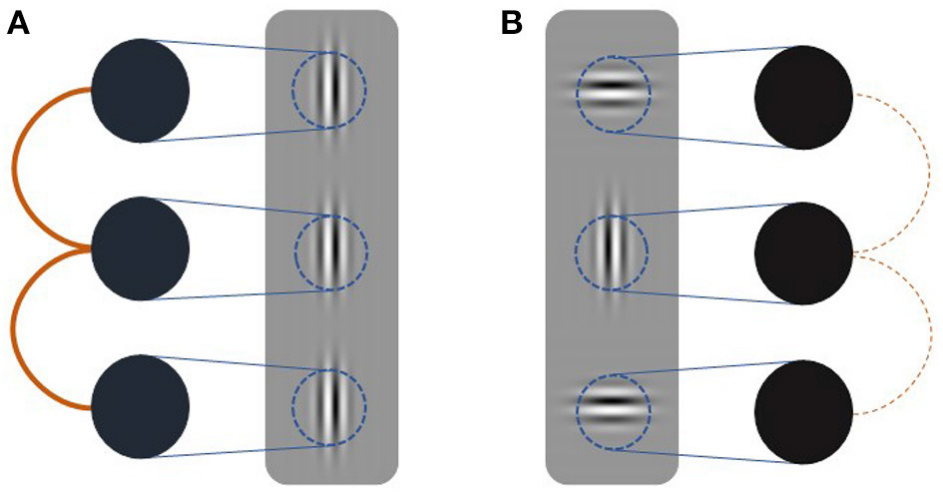
Schematic of simulation setup. Three oscillators (black circles) receive input (blue lines) dependent on the contrast presented in their receptive fields (blue circles). Coupling strength (orange lines) is dependent on the relative orientation of the target and flanker Gabor patches. Thick solid lines indicate strong coupling whereas thin dashed line indicate weak coupling. **(A)** Coupling between oscillators receiving collinear inputs is strong. **(B)** Coupling between oscillators receiving orthogonal input is weak.

We formalize this conjecture using a simple recurrent V1 model of three vertically arranged neural populations encoding a target and two flanker Gabor patches (Figure 2). Each neural population is modeled as a Kuramoto oscillator whose intrinsic frequency is contrast-dependent with the exact relationship being modulated by attention. Connectivity between oscillators depends on the degree to which flanker orientations are coaxial with the group's collinear arrangement. To test whether surround effects in the model mimic neural V1 responses, we employ a flanker-target configuration akin to Polat et al. (1998) and vary contrasts systematically. Our model exhibits contrast and orientation dependent facilitative and suppressive flanker effects in agreement with behavioral and neurophysiological findings. Furthermore, attention modulations of the frequency-contrast relation allow our model to account for known attention effects on collinear facilitation (Freeman et al., 2001, 2003).

## 2. Methods

### 2.1. Oscillator Model

We model the neural populations corresponding to vertically adjacent, non-overlapping, receptive fields as weakly-coupled oscillators of the Kuramoto type (Kuramoto, 1984). Several studies have shown that the Kuramoto model well captures the oscillatory behavior emerging in populations of spiking neurons (Bhowmik and Shanahan, 2012; Politi and Rosenblum, 2015; Lowet et al., 2017). The phase of model populations evolve according to

(1) $$\begin{array}{l} {\dot{\theta }}_{i}=\omega_{i}+\frac{K}{N}\overset{N}{\underset{j=1}{\sum }}\sin\left(\theta_{j}-\theta_{i}\right) \end{array}$$

where *N* = 3 is the number of neural populations (target and two flanking Gabors), *K* is the coupling strength between populations and ω_*i*_ is the intrinsic angular velocity of population *i*. Note that we do not include noise in the model as it has been shown to mainly increase the critical coupling strength and only minimally affects qualitative synchronization behavior (Sakaguchi, 1988). We confirm this by repeating a number of simulations with noise (see Supplementary Figure 2). In our model the coupling strength captures collinearity as lateral connections between neural populations in early visual cortex have been shown to depend on their relative orientation tuning (Ts'o et al., 1986; Gilbert and Wiesel, 1989; Bosking et al., 1997; Schmidt et al., 1997). Angular velocity ω is related to frequency by ω = 2π*f* and hence reflects stimulus contrast. We estimate the exact contrast-frequency relationship from electrophysiological data previously obtained from two awake macaque monkeys. The data, which was published previously (Roberts et al., 2013), contains peak frequencies of local field potentials (LFPs) measured in V1 and V2 populations in response to grating stimuli of various contrasts. We describe this empirical contrast-frequency relationship with a sigmoid function:

(2) $$\begin{array}{l} f=\frac{\gamma }{1+\exp\left(-\beta c+\alpha \beta \right)} \end{array}$$

where *c* is the contrast and α = 10.74, β = 0.057 and γ = 44.77 (Figure 1C) are the best fitting inflection point, slope and gain.

### 2.2. Simulation Experiments

To investigate whether the synchronization mechanism can account for empirical effects of collinear alignment of the target and flanker ensemble and of target contrast, we simulate the system of WCOs for a range of coupling values and target contrasts while keeping flanker contrast fixed. A range of coupling values is used which best reflects the dynamic range of the model.

To investigate the effect of attention, we repeat simulations with attention directed either at the target or the flankers. We model attention as an increase in response gain (γ) of the sigmoidal contrast-frequency function (Joon Kim et al., 2007; Hermann et al., 2010; Ferguson and Cardin, 2020) of the attended oscillator. Specifically, we use γ = 49 for attented oscillators. This small increase of γ is sufficient to show how attention can shift the switching point of facilitation to suppression and that the attention mechanism can account for empirical findings.

The Kuramoto order parameter (*r*) is used as a measure of synchrony among the three oscillators (target and two flankers; Acebrón et al., 2005):

(3) $$\begin{array}{l} r=\frac{1}{N}\overset{N}{\underset{j=1}{\sum }}e^{i\theta_{j}} \end{array}$$

The mean effective frequency of each oscillator is:

(4) $$\begin{array}{l} \bar{f_{i}}=\frac{\langle {\dot{\theta }}_{i}\rangle }{2\pi } \end{array}$$

where $\langle {\dot{\theta }}_{i}\rangle$ is the arithmetic mean of changes in the phase of oscillator *i* recorded from our simulations. The first 99 time steps are discarded when computing the arithmetic mean to remove transients and focus on equilibrium dynamics.

In all simulations we use the forward Euler integration method to update the phases of the oscillators, a simulation time of 1 *s* and a time step Δ*t* = 0.002 *s*. Each simulation is initialized with random phases, the experiments are repeated 50 times and results are averaged.

## 3. Results

### 3.1. Experiment 1: Facilitation and Suppression

**Figure 3:**
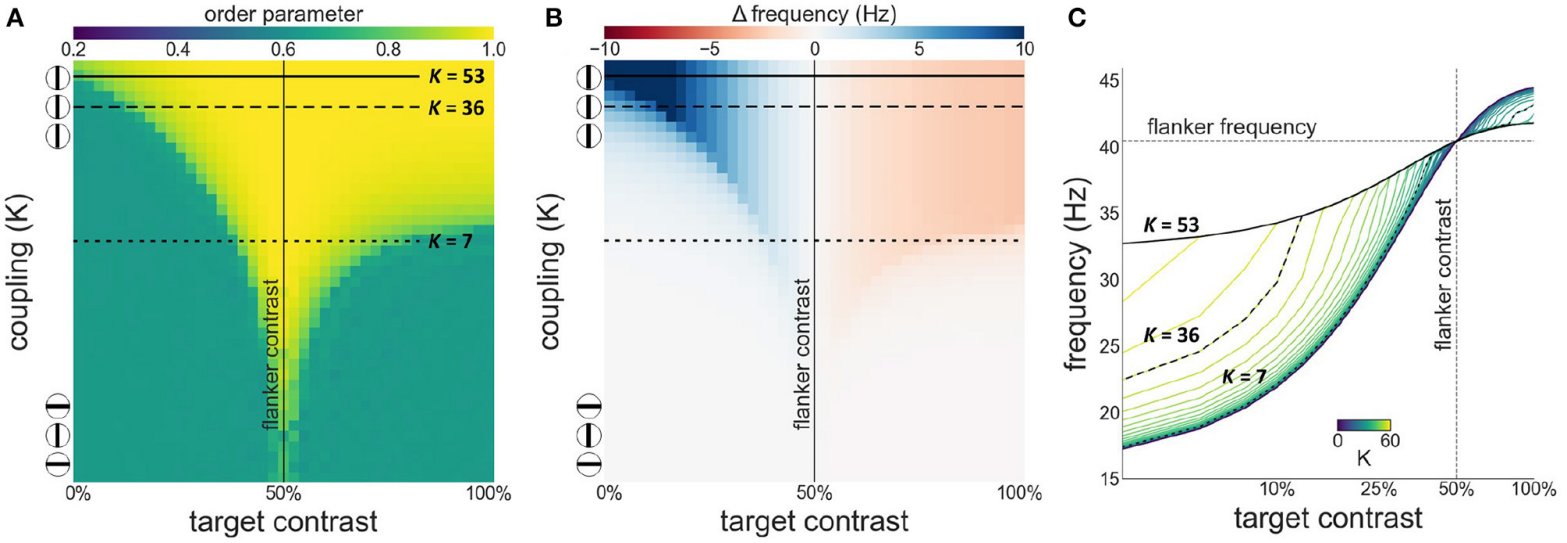
Interactions between target and flanker populations. Flanker contrast is fixed at 50% (vertical line) while target contrast varies (x-axis). The three encircled lines and the top and bottom of the y-axis in **(A,B)** reflect the relation of the coupling strength (*K*) to collinearity of the target to the flankers. In this and subsequent Figures 4, 7: **(A)** Phase coherence among the three populations as a function of target contrast and coupling strength. **(B)** The difference between the observed (mean effective frequency) and intrinsic frequency of the target oscillator as a function of target contrast and coupling strength. Reflects facilitation and suppression of frequency. **(C)** The firing frequency plotted for various target contrasts and coupling values (*K*). Firing frequency of the horizontal black lines in **(A,B)** is represented by the highlighted black lines in **(C)**.

We first investigate synchronization as well as facilitation and suppression effects, as target contrast is varied while flanker contrast is fixed at 50 % (see Figure 3). Figure 3A shows that there is a wide, asymmetric, synchronization region for *K* ≥ 7. Asymmetry is the result of the sigmoidal relationship between contrast and intrinsic frequency. Since intrinsic frequency begins to saturate at approximately 60 % contrast, frequency differences between target and flankers are small for target contrasts ≥50 % allowing for synchronization to occur for large contrast differences. Saturation of the contrast-frequency relationship also manifests itself as asymmetric facilitation and suppression effects (Figure 3B). Large facilitation effects occur for target contrasts <50 % where target intrinsic frequencies differ greatly from the flanker's intrinsic frequency (quasi-linear portion of the contrast-frequency relationship). Smaller suppression effects occur for target contrasts >50 % as target intrinsic frequencies remains close to the flanker's intrinsic frequency due to saturation of the contrast-frequency relationship. Figure 3C shows the absolute frequency of the target oscillator with facilitation and suppression effects in a format similar to Figures 2a,b in Polat et al. (1998). These modulatory effects are robust to noise (see Supplementary Figure 2).

**Figure 4:**
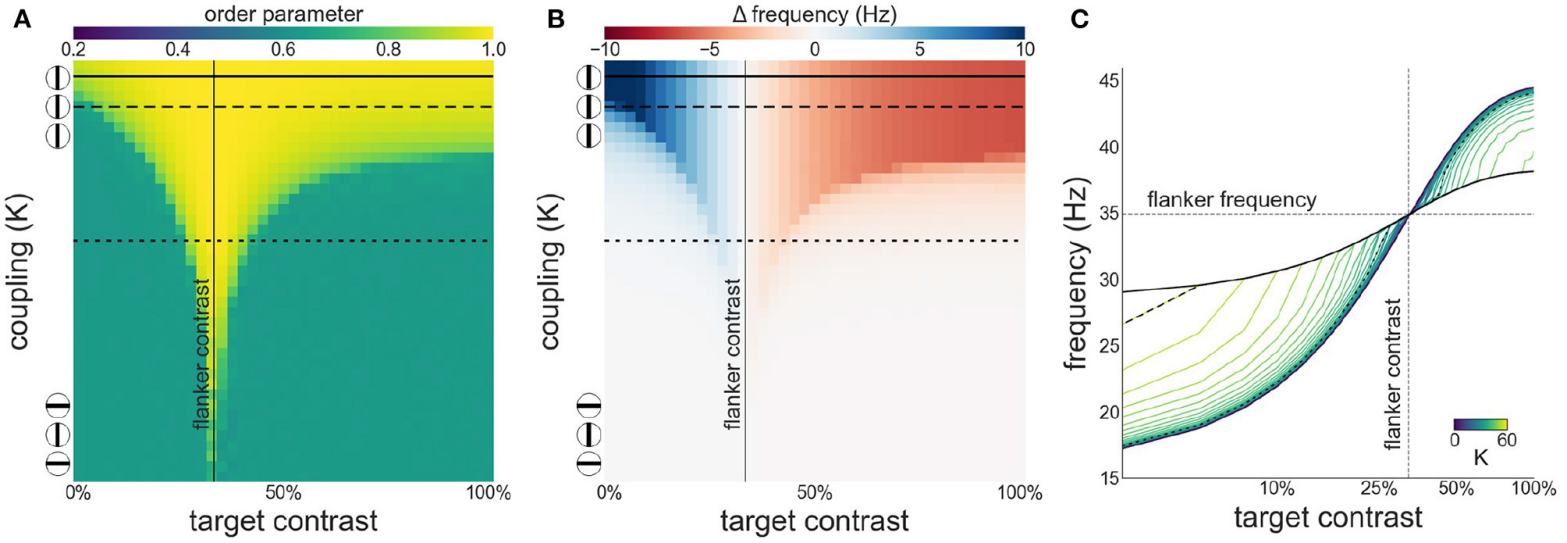
Interactions between target and flanker populations, with flanker contrast fixed at 33% (vertical line). Formatting is identical to Figure 3: **(A)** Arnold tongue **(B)** difference mean effective frequency and intrinsic frequency of target **(C)** firing frequency of the target.

Next, we investigate both synchronization as well as facilitation and suppression effects, as target contrast is varied while flanker contrast is fixed at 33 % (see Figure 4). This is the contrast value at which intrinsic frequency reaches ~80 % of its asymptotic value (γ) given by our contrast-frequency curve. This corresponds to the flanker contrast used in Polat et al. (1998) in relative terms (with respect to the neurons' contrast response function). We observe a similar asymmetry as seen in the configuration with 50 % flanker contrast. However, the target frequency is facilitated for contrast levels up to 33 %. Because of the saturation of the intrinsic frequency beyond 60 %, the suppression is stronger at target contrast beyond 33 %.

### 3.2. Experiment 2: Facilitation and Suppression With Attention

It is well-established that the contrast-frequency relationship is modulated by attention (Ito and Gilbert, 1999; Freeman et al., 2001, 2003; Giorgi et al., 2004; Khoe et al., 2006). Here, we model attention as a response gain factor that scales the sigmoid function and investigate the effects of attention directed toward the target or the flankers.

**Figure 5:**
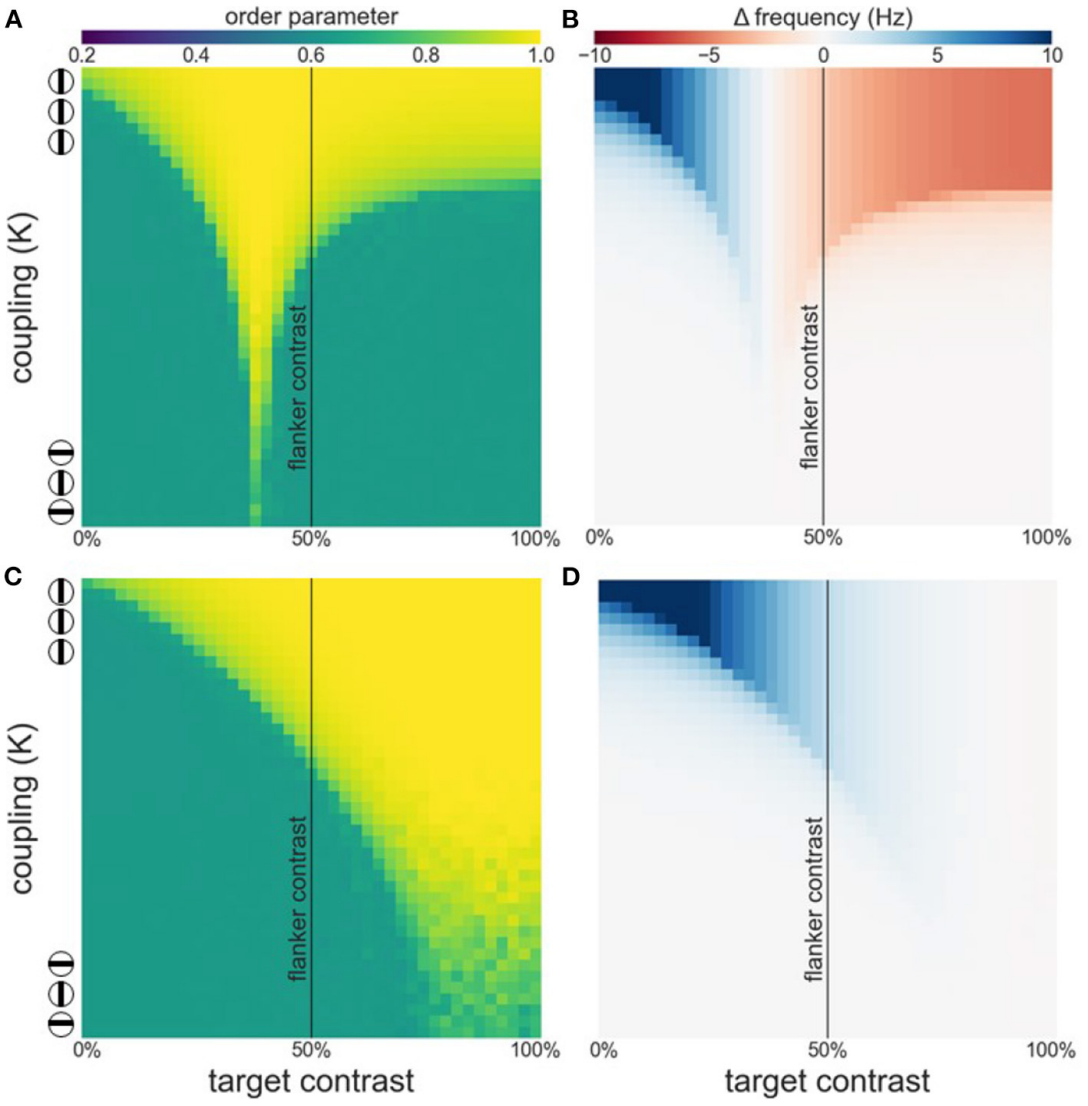
Effects of attention on phase coherence **(A,C)** and frequency difference **(B,D)**. **(A,B)** Attention on the target. **(C,D)** Attention on the flankers. Flanker contrast is fixed at 50% (vertical line).

As can be appreciated from Figures 5A,B attentional modulation of the target causes a left-shift of the synchronization region and the facilitation-suppression switching point. As the maximum of the contrast-frequency curve of the target population has increased, its intrinsic frequency surpasses that of the flankers at lower contrast values. This gives rise to the observed shift. Furthermore, though the response of all populations still saturates at approximately 60 % contrast, the increased response of the attended target ensures that intrinsic frequencies of target and flankers are sufficiently different for strong suppression effects to occur.

**Figure 6:**
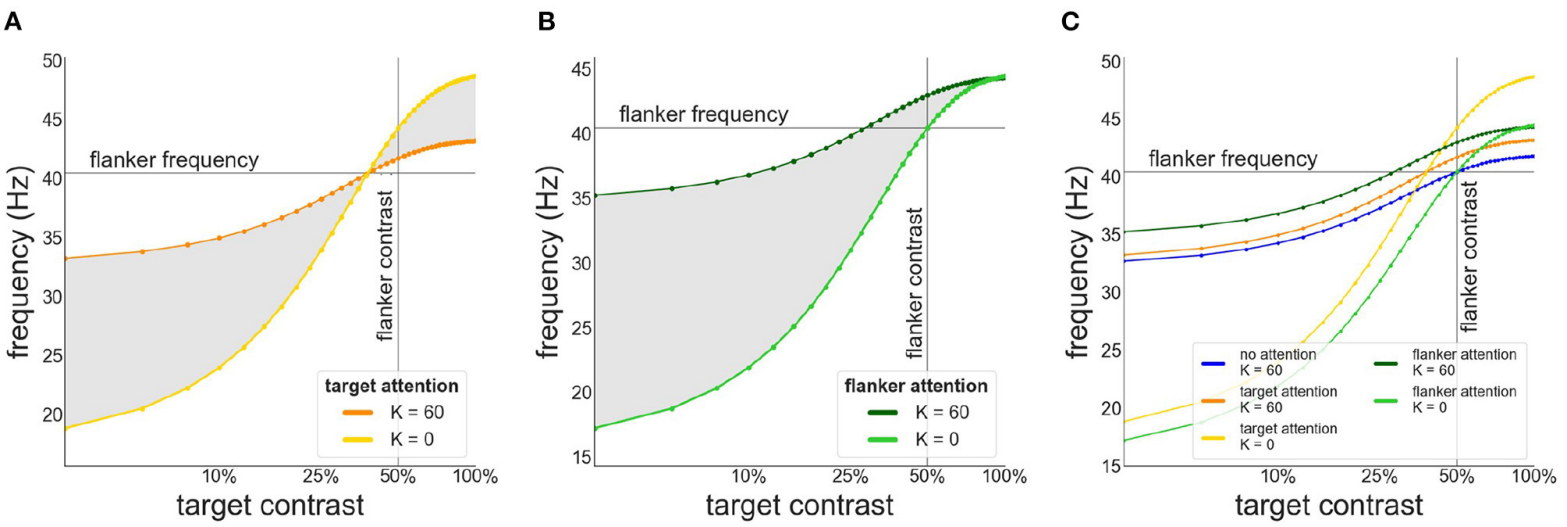
Target frequency as a function of contrast in the presence of attention. **(A)** Attention directed toward the target. Frequency of target patch is shown in the absence (yellow) and presence of collinear (orange) flankers **(B)** Attention directed toward the flankers. Frequency of target patch in absence (light green) and presence (dark green) of collinear flankers. **(C)** Comparison of the target frequency for different attention configurations. Target frequency is shown without attention (blue) or when attention is directed toward the target (orange/yellow) or the flankers (green).

**Figure 7:**
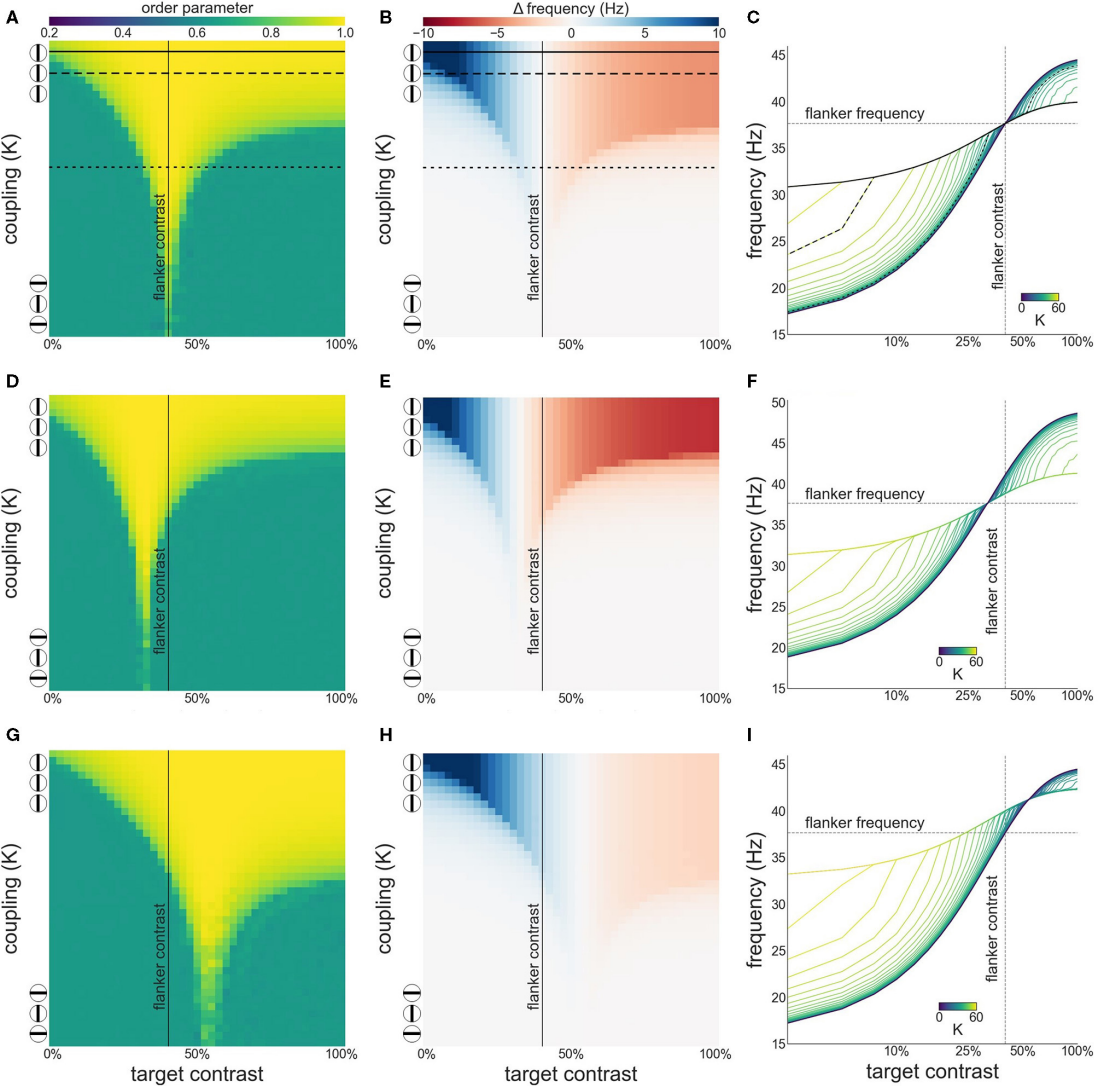
Interactions between target and flanker populations, with flanker contrast is fixed at 40% (vertical line). Formatting of each row is identical to Figure 3. Results are shown for the simulation without attention **(A–C)**, with attention on the target **(D–F)**, and on the flankers **(G–I)**.

When attention is directed at the flankers there is a general increase of the facilitation at lower target contrast levels and a corresponding reduction in suppression at higher contrast levels (Figures 5C,D). Depending on the overall strength of attention, suppression may disappear entirely (Figure 5D). Interestingly, attending the flankers causes larger facilitation than attending the target, an effect that is particularly pronounced at low target contrasts (Figure 6). This is in line with observations made by Freeman et al. (2001). However, these authors performed experiments with 40 % flanker contrast. To show that our findings hold true with these flanker contrasts as well, we repeated our simulations with 40 % flanker contrast. Without attention, suppression is more pronounced compared to flankers with 50 % contrast (see Figures 3, 7A–C). This is because the contrast-frequency relation is not yet saturated at 40 % (Figure 1C). Attention directed toward the flankers decreases suppression and increases facilitation (Figure 7I), in line with results from Freeman et al. (2001).

### 3.3. Experiment 3: Varying Flanker-Flanker, Flanker-Target Coupling Ratio

**Figure 8:**
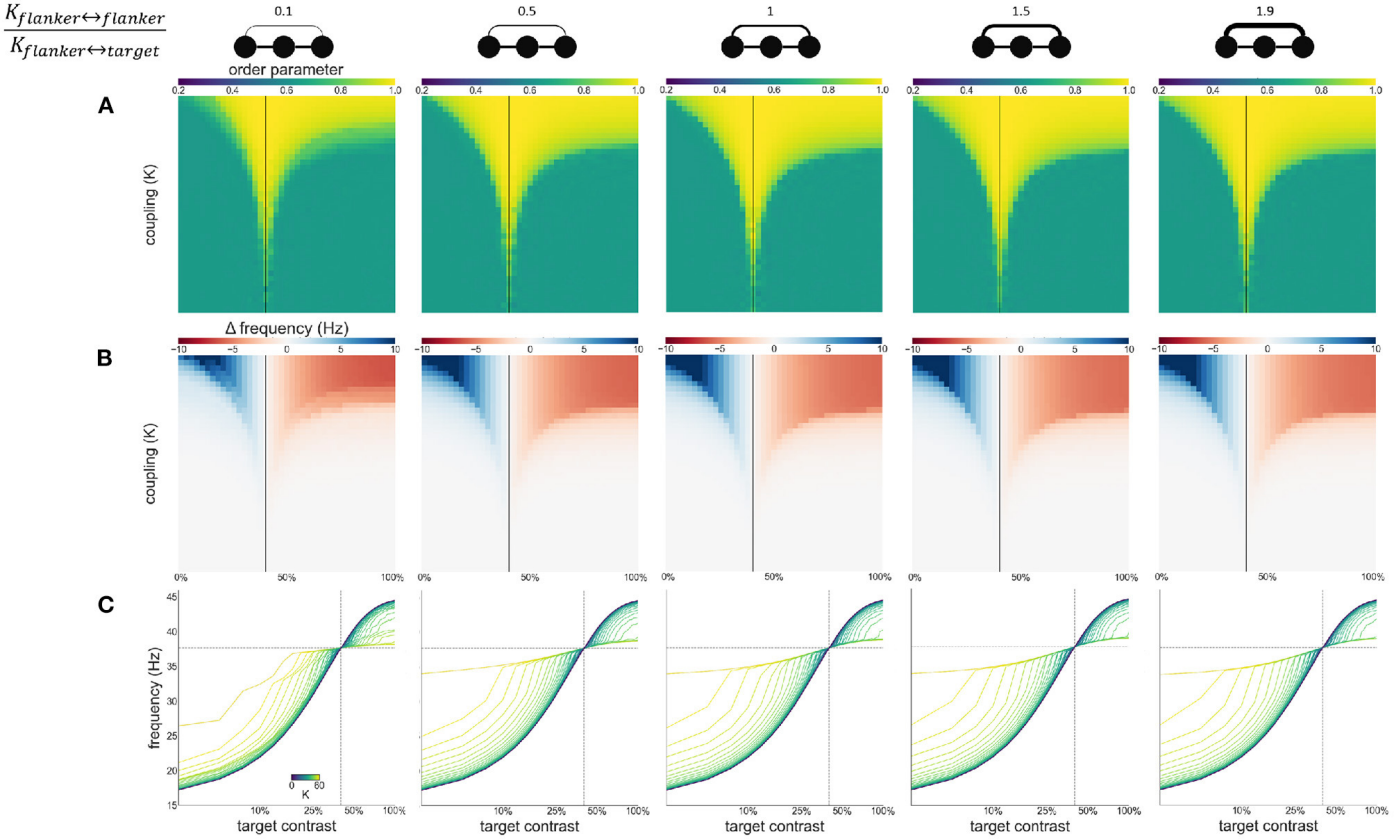
Effects of varying flanker-flanker coupling ratio. Ratio increases from left to right. Ratios are given at the top of the figure together with illustrations of the relevant connections. Formatting of each column is identical to Figure 3: **(A)** Arnold tongue **(B)** difference mean effective frequency and intrinsic frequency of target **(C)** firing frequency of the target.

To explore the effect of variable asymmetric coupling between flanker and target populations we varied the coupling strength in two ways. First, the coupling ratio between flankers is varied: $\frac{K_{flanker↔flanker}}{K_{flanker↔target}}$. Specifically, the coupling strength between the flankers is systematically varied while the coupling between flankers and the target is kept constant. The constant coupling strength *K* is the same value as in previous experiments. The varying coupling strength is obtained by multiplying this value *K* by the desired coupling ratio. The Arnold tongue (Figure 8A) and the facilitation and suppression plots (Figure 8B) are similar for the various ratios presented here. Only when the ratio is 0.1, the synchronization, facilitation and suppression start to deviate from the other simulations for large *K*. This might be due to the flankers having low coupling and therefore they have a decreased tendency to synchronize with each other. This likely causes the target to synchronize at an instance with one of the flankers, but not as easily with both. As a result, for larger differences in intrinsic frequency between the flankers and the target, the 3 oscillators do not synchronize all together but more often with only 2 oscillators (target and 1 flanker) at a time, resulting in more chaotic behavior (Figure 8C).

**Figure 9:**
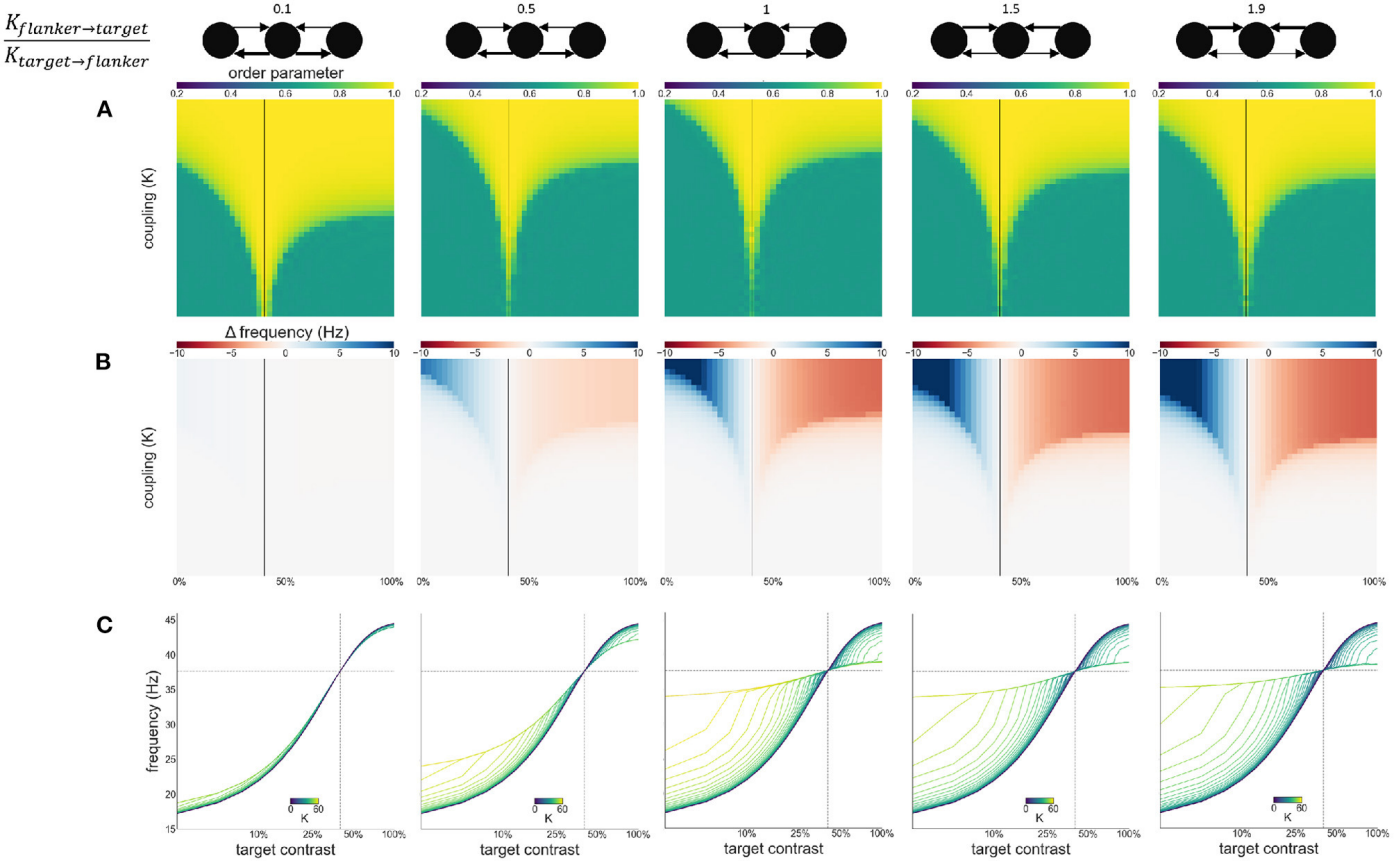
Effects of varying directed coupling ratio between targets and flankers. Ratio increases from left to right. Ratios are given at the top of the figure together with illustrations of the relevant connections. Formatting of each column is identical to Figure 3: **(A)** Arnold tongue **(B)** difference mean effective frequency and intrinsic frequency of target **(C)** firing frequency of the target.

Second, we modulate the directed coupling strength to and from the target and flankers: $\frac{K_{flanker\to target}}{K_{target\to flanker}}$. Flanker to flanker coupling is kept constant at *K*. The flanker-to-target, and target-to-flanker coupling are varied by increasing or decreasing them relative to *K* (multiplying or dividing by the desired ratio). Asymmetric coupling schemes (ratio higher or lower than 1) result in a larger Arnold tongue area (Figure 9A). This is in particular the case when target-to-flanker coupling is larger than flanker-to-target coupling. This effect is caused by the either the target dominating the flankers (at the low ratio), or flankers dominating the target (at the high ratio). That is, the target oscillator has to adjust the frequency less to the flanker oscillators if it dominates the flankers, and more if it is dominated by the flankers (Figures 9B,C).

## 4. Discussion

We investigated whether synchronization and desynchronization among weakly coupled oscillators can account for contextual modulation in early visual cortex. More specifically, we explored the possibility that a synchronization mechanism might underlie collinear facilitation and suppression. Synchrony among weakly coupled oscillators depends on their intrinsic frequencies as well as the strength of their coupling. Contrast-dependent local firing frequency and the dependence of lateral connectivity on orientation tuning provide the neurophysiological and anatomical ingredients to account for collinear facilitation and suppression effects. Neural populations encoding the target and flanker have varying firing frequency dependent on the bottom-up input they receive. Additionally, these populations are mutually coupled, which allows them to synchronize. Depending on the relative intrinsic frequency of the target and flanker populations, their synchronization leads to either *slowing down* or *speeding up* of the effective frequency of the target population.

Using this mechanism we were able to qualitatively replicate empirical findings of Polat et al. (1998) and Freeman et al. (2001). Specifically, we show that the proposed mechanism can account for a switch from facilitation to suppression as target contrast approaches flanker contrast (Polat et al., 1998). Furthermore, implementing an attentional gain allowed us to replicate the findings of Freeman et al. (2001); namely that attending the flankers causes larger facilitation than attending the target and that this effect is particularly pronounced for low target contrasts (Freeman et al., 2001). Note that we chose to simulate attention as a response gain rather than contrast gain. This choice is based on (a) empirical and modeling results showing that if attention is directed toward the full size of a receptive field, it manifests as a response gain (Hermann et al., 2010), (b) that gratings used by both Polat et al. (1998) and Freeman et al. (2001) encompass the full size of receptive fields of V1 neurons and (c) that attention is directed toward the full grating rather than merely a portion of it (Freeman et al., 2001). While our results agree with the majority of observations reported previously, we could only replicate Polat et al. (1998)'s observation that a switch from facilitation to suppression occurs when target contrast is smaller than (rather than equal to) flanker contrast when we directed attention at the target. Given that Polat et al. (1998) conducted their experiments in anesthetized cats, it is unlikely that attention accounts for the position of the switch in their results.

In our model, a switch from facilitation to suppression occurs when the intrinsic frequencies of target and flanker populations are equal. In the absence of attention gain, this occurs when their respective contrasts are equal. We modeled attention as a response gain which allows attended populations to exhibit higher frequencies even though contrast levels are equal. It is the resulting difference in the contrast-frequency relationship between target and flanker populations that accounts for the leftward shift of the switching point between facilitation and suppression toward lower target contrasts (Figure 6). It is possible that other factors besides attention lead to different contrast-frequency relationships in the three populations. One is simply natural variation in this relationship across neurons. However, this would imply that the location of the switch observed by Polat et al. (1998) is incidental and could equally have occurred when target contrast is larger than flanker contrast, had they measured other neurons. A more likely scenario is that local network interactions are responsible for differences in the contrast frequency relationships. In the absence of external stimulation neuronal populations exhibit strong power in the delta to alpha frequency ranges, especially if the animal is anesthetized (Sellers et al., 2013). These background populations may act as a periodic force on the stimulated populations. If this force is sufficiently strong, it might suppress the frequencies exhibited by the stimulated populations. Given the distance dependence of lateral connections, the flanker populations may be more strongly influenced by the background than the target population (which is enclosed by the flankers), leading to distinct frequencies of the two populations even when their contrast input is equal (see Supplementary Figure 1). Furthermore, we estimated the contrast-frequency relationship from neurophysiological recordings in *awake* monkeys. Given that contrast-frequency relationship differs for awake and anesthetized animals (Sellers et al., 2015), this might have additionally contributed to differences in contrast dependencies between our and Polat et al. (1998)'s observations. This would be in line with previous findings observing changes in synchronization behavior of the visual cortex following brain state modulations. For example, sleep is known to influence cortical oscillations and might have an effect on local gamma synchronization in the visual cortex (Adamantidis et al., 2019). We varied the coupling between target and flanker populations asymmetrically in two ways. These variations on the coupling scheme mainly affect the area of the Arnold tongue (Figures 8A, 9A) and the strength of the facilitation and suppression effects (Figures 9B,C). These results show that for variable coupling between populations, the main conclusions hold. We do not study the effects of coupling variability within populations as we do not model individual neurons.

We make use of a simple Kuramoto model to simulate WCOs. This model allows us to focus on the essential principles of the oscillation mechanism. A model that abstracts away the details of interconnected spiking neurons and leaving us with the aspects that we hypothesize are mechanistically relevant allows us to truly evaluate the proposed components of our mechanism. Because some behavior might be the result of some specific detail rather than global principles, including more details about the underlying circuit would make it harder to ascribe explanatory power to what we find relevant (i.e., oscillations, intrinsic frequency, coupling).

Several studies have shown that Kuramoto neural mass models capture the oscillatory and synchronization behavior of networks of spiking neuron populations. Bhowmik and Shanahan (2012) showed that neural networks of quadratic integrate-and-fire neurons and Hodgkin-Huxley neurons exhibited similar behavior to networks of Kuramoto oscillators. Politi and Rosenblum (2015) obtained similar results comparing models of leaky-integrate-and-fire neurons and Winfree-type ensembles of oscillators to a Kuramoto network model. Lowet et al. (2017) showed that behavior of a coupled pyramidal-interneuron-gamma-network (PING) implemented with Izhikevich neurons could be fully reproduced with a Kuramoto model, and therefore reproduce neurophysiological findings. These findings support the use of the Kuramoto model for investigation of neural oscillations and synchrony.

Prior modeling studies have suggested other mechanisms besides local synchronization to explain collinear facilitation and suppression effects. Somers et al. (1998), for instance, presented a model of V1 that explains the switch from collinear facilitation to collinear suppression through local lateral connections among excitatory and inhibitory neurons. Specifically, inhibitory neurons in their model exhibit a steeper slope of the frequency-current curve as well as higher functional thresholds than excitatory neurons. Together, these properties of inhibitory neurons ensure that the ratio of local excitatory currents to local inhibitory currents evoked by a stimulus is high for low stimulus contrast and low for high stimulus contrast. In conjunction with long-range interactions between populations encoding target and flanker stimuli, this mechanism can account for the switch from facilitation to suppression as target contrast increases. However, in contrast to our model which accounts for these effects with a general synchronization mechanism, the model of Somers et al. (1998) appears to be specifically tailored to this specific effect. De Meyer and Spratling (2009) presented a model of attentional gating of collinear facilitation. In their model, collinear facilitation is the result of long-range, excitatory, lateral connections (due to a lack of long-range lateral inhibition the model does not account for collinear suppression). Attention, operating via cortical feedback connections, then gates the effects of these long-range connections. Specifically, gating in their model is the result of non-linear dendritic interactions between inputs arriving on different parts of the dendritic tree of cortical pyramidal cells. The attentional gating signal into V1 originates from a competition between neuron populations in extrastriate areas V2 and V4 which may itself be biased by an attentional feedback signal from frontal cortex. With this mechanism, De Meyer and Spratling (2009) could account for modulatory effects of attention reported by Ito and Gilbert (1999). Interestingly, their model predicts that attention on the target leads to the largest gain in facilitation whereas our model predicts that the largest gain can be achieved by attending the flankers. Since the two models have been evaluated against different experimental designs and stimulus setups, it could be worthwhile to conduct empirical studies explicitly testing the diverging predictions of these two models.

It is likely that the visual system exploits both temporal and rate codes (Kiper et al., 1996; Biederlack et al., 2006; Montemurro et al., 2008). For instance, Kiper et al. (1996) have shown that figure-ground segregation and figure grouping may occur in the absence of neural synchrony when figure and ground elements exhibit temporal flickering at distinct phases. This indicates that neural synchrony may not be a general mechanism for binding leaving room for a potential rate-based mechanism. However, it is difficult to infer the synchronization behavior of neural populations from purely psychophysical experiments. Phases of neural oscillators representing the figure and background may have been more strongly driven by local interactions than by the phase of the stimulus flicker. Differences in intrinsic frequencies resulting from stimulus properties other than flicker such as the orientation of texture elements may thus still have lead to desynchronization between figure and background. In this case, the synchronization mechanism proposed here could still form the basis of the local binding effect. It would be interesting to simulate the experiments of Kiper et al. (1996) using our mechanism in conjunction with a periodic forcing signal reflecting stimulus flicker.

To our knowledge, we provide a first mechanistic interpretation of how facilitation and suppression of interacting circuits and perceptual grouping may emerge through neural synchrony. Our findings are in line with several studies ascribing a functional role to local synchronization such as contour integration (Li, 1998), texture discrimination (Baldi and Meir, 2008) and figure-ground segmentation (Yamaguchi and Shimizu, 1994). Li (1998) presents a computational model which is capable of performing contour integration and shows that units representing contour elements are synchronized while these elements are desynchronized with background elements. Other studies use synchronization of WCOs to explain other psychophysical grouping effects like texture discrimination (Baldi and Meir, 2008) and figure-ground segmentation (Yamaguchi and Shimizu, 1994). Alais et al. (1998) first discovered psychophysical evidence of a correlation between feature binding and the temporal correlation of neuronal firing patterns. These studies highlight the functional role of synchronization in early visual processing.

In light of findings that gamma oscillations are ubiquitous in the cortex (Buzsáki and Wang, 2012) and that synchrony is feature specific (Lowet et al., 2017), a functional role for synchronization among neuronal populations has been proposed (Lowet et al., 2015, 2017, 2018). Our model provides additional support for this theory, as it can straightforwardly account for collinear facilitation and suppression effects. Future work is needed to expand this framework to further perceptual effects, both within (e.g., surface perception) as well as beyond (e.g., auditory perception) the visual domain; and to develop more sophisticated, biophysical, models of neural oscillators, and their interactions.

## Data Availability Statement

The data analyzed in this study is subject to the following licenses/restrictions: The data that support the findings of this study were made available by Mark Roberts upon request. Restrictions apply to the availability of these data, which were used under license for this study. Requests to access these datasets should be directed to mark.roberts@maastrichtuniversity.nl.

## Author Contributions

KE and MS: conceptualization of the study. KE: modeling and performing experiments. KE, MS, and JP: writing and editing article. All authors contributed to the article and approved the submitted version.

## Conflict of Interest

The authors declare that the research was conducted in the absence of any commercial or financial relationships that could be construed as a potential conflict of interest.

## Publisher's Note

All claims expressed in this article are solely those of the authors and do not necessarily represent those of their affiliated organizations, or those of the publisher, the editors and the reviewers. Any product that may be evaluated in this article, or claim that may be made by its manufacturer, is not guaranteed or endorsed by the publisher.

## Resource Identification Initiative

Python Programming Language, RRID:SCR_008394.
