# Supplementary material for: Cortical Synchrony as a Mechanism of Collinear Facilitation and Suppression in Early Visual Cortex
Source: Front Syst Neurosci. 2021 Jul 29;15:670702. doi: 10.3389/fnsys.2021.670702 (PMC8358273; doi:10.3389/fnsys.2021.670702)
Supplement: Supplementary file 1 [file Data_Sheet_1.pdf]

Supplementary Material

1 FOURTH BACKGROUND OSCILLATOR

A background population of neurons is added as a fourth oscillator with fixed 10 Hz frequency. The fourth oscillator is connected to the target and flanker oscillators with a coupling strength of 2/3L and L respectively to simulate differing proportions of background inputs received by the three (one target and two flanker) populations. Results are shown for L=60 and L=100 (Figure S1). Low-frequency background input causes a left shift of the facilitation-suppression switching point.

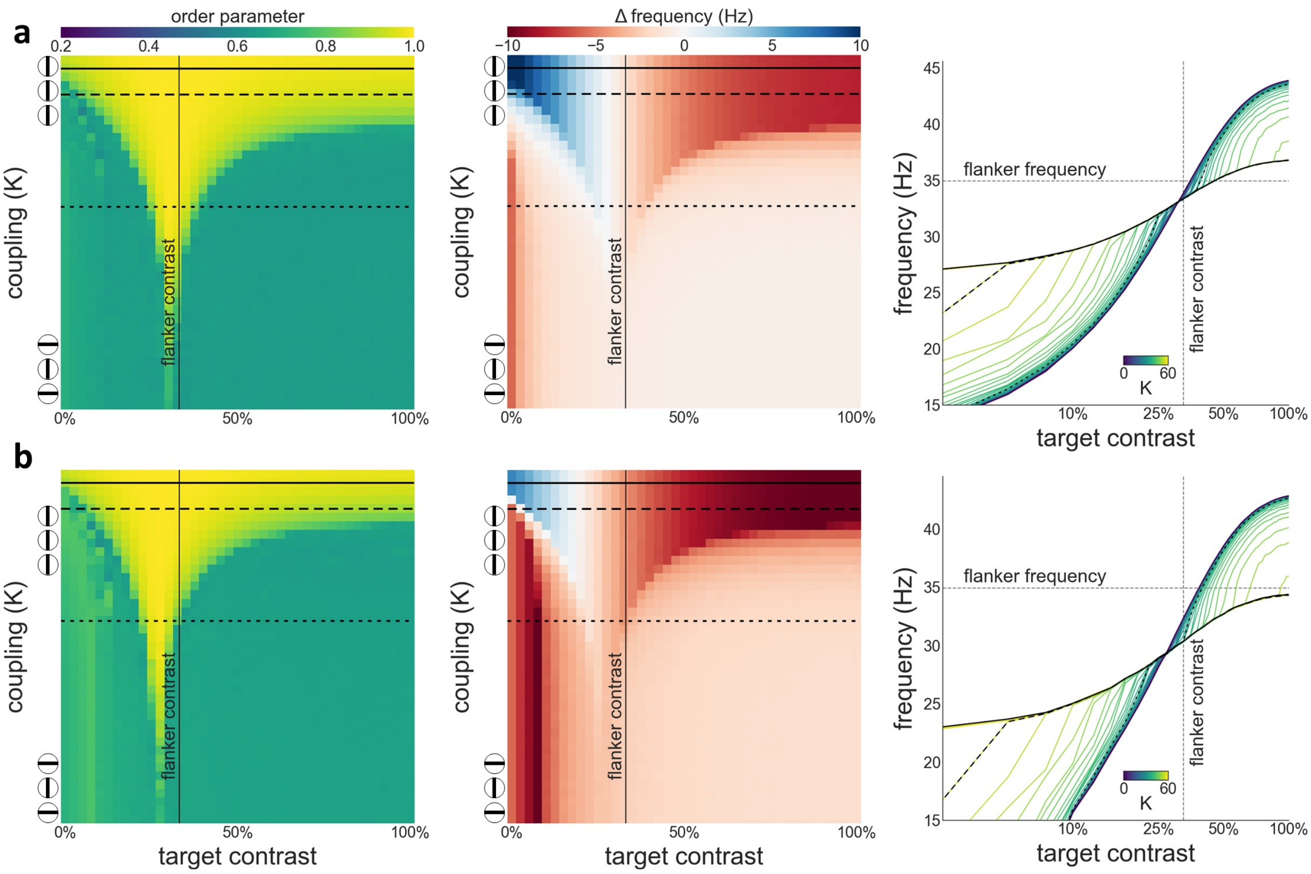

Figure S1. A fourth oscillator fixed at a frequency of 10Hz and a connection strength of L=60 (a) or L=100 (b) to the flankers and 2/3L to the target causes a left shift of the suppression-facilitation switching point.

2 NOISE

Simulations including a noise term in the Kuramoto function:

$$\dot{\theta}_i = \omega_i + \boldsymbol{\xi_{i,t}} + \frac{K}{N}\sum_{j=1}^{N}\sin(\theta_j - \theta_i) \qquad \text{(S1)}$$

Pink noise with a scaling exponent of 1 is used similar to Lowet et al. (2015). Simulations with different noise levels are shown in Figure S2

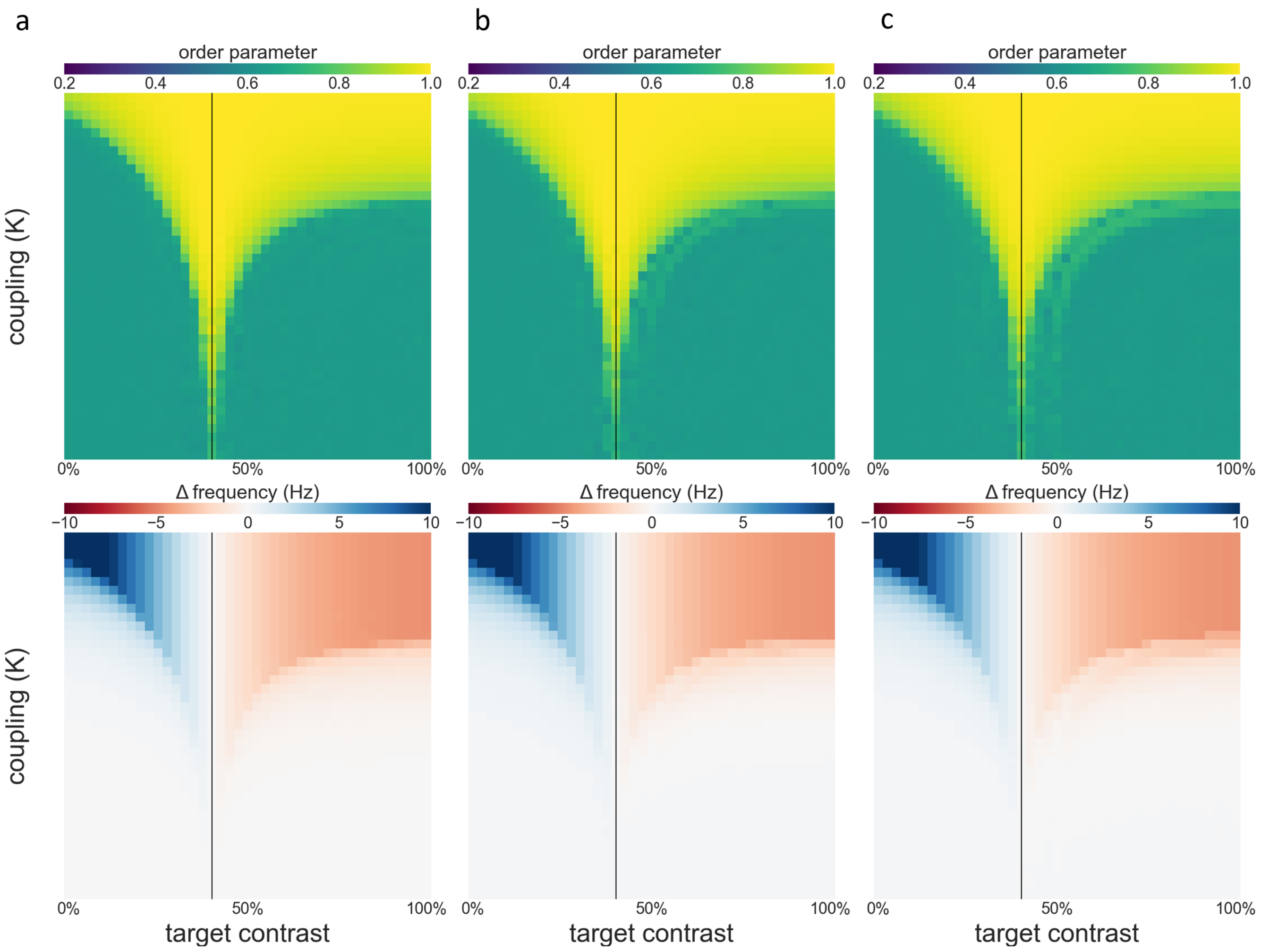

Figure S2. Model simulations with various noise levels; (a) no noise, (b) $0.5 * \xi$ and (c) $1 * \xi$ the noise term.

REFERENCES

Lowet E, Roberts M, Hadjipapas A, Peter A, van der Eerden J, De Weerd P. Input-dependent frequency modulation of cortical gamma oscillations shapes spatial synchronization and enables phase coding. *PLoS Computational Biology* **11** (2015). doi:10.1371/journal.pcbi.1004072.
